# Receptor-interacting protein kinase 2 promotes triple-negative breast cancer cell migration and invasion via activation of nuclear factor-kappaB and c-Jun N-terminal kinase pathways

**DOI:** 10.1186/bcr3629

**Published:** 2014-03-19

**Authors:** Stina M Singel, Kimberly Batten, Crystal Cornelius, Gaoxiang Jia, Gail Fasciani, Summer L Barron, Woodring E Wright, Jerry W Shay

**Affiliations:** 1 Departments of Cell Biology, University of Texas Southwestern Medical Center, 5323 Harry Hines Boulevard, Dallas, TX 75390-9039, USA; 2 Departments of Internal Medicine, University of Texas Southwestern Medical Center, Dallas, TX, USA

## Abstract

**Introduction:**

Metastasis is the main cause of breast cancer morbidity and mortality. Processes that allow for tumor cell migration and invasion are important therapeutic targets. Here we demonstrate that receptor-interacting protein kinase 2 (RIP2), a kinase known to be involved in inflammatory processes, also has novel roles in cancer cell migration and invasion.

**Methods:**

A total of six breast cancer expression databases, including The Cancer Genome Atlas, were assessed for *RIP2* expression among various clinical subtypes and its role as a prognostic biomarker. mRNA fluorescence *in situ* hybridization (FISH) for *RIP2* was performed on 17 stage III breast cancers to determine if there was a correlation between *RIP2* expression and lymph node involvement. RNA-interference was used to knock-down *RIP2* expression in MDA-MB-231, Htb126, SUM149PT, MCF7, T47D, and HCC1428 cells. Cell migration and invasion were measured *in vitro* by scratch/wound healing and transwell migration assays. A xenograft mouse model was used to assess tumor growth and chemosensitivity to docetaxel *in vivo* in MDA-MB-231 cells with and without *RIP2* small hairpin RNA knockdown. Western blot and immunofluorescence imaging were used to evaluate protein expressions.

**Results:**

Interrogation of expression databases showed that *RIP2* expression is significantly over-expressed in triple-negative breast cancers (TNBC: estrogen-receptor (ER) negative, progesterone-receptor (PR) negative, Her2/neu- (Her2) negative), compared to other clinical subtypes. High *RIP2* expression correlates with worse progression-free survival using a combined breast cancer expression array dataset consisting of 946 patients. Multivariate analysis shows *RIP2* as an independent prognostic biomarker. Knock-down of *RIP2* significantly decreases migration in both scratch/wound healing and transwell migration assays in MDA-MB-231, Htb126, SUM149PT, MCF7, and T47D cells and is correlated with decreased Nuclear Factor-kappaB and c-Jun N-terminal kinase (JNK) activation. Finally, *RIP2* knock-down leads to increased sensitivity to docetaxel and decreased tumor mass and lung metastases in a xenograft mouse model.

**Conclusion:**

These results highlight RIP2 as a pro-metastasis kinase in patients with advanced breast cancer. These results also illustrate a novel role for this kinase in addition to its known role in inflammation, and suggest that targeting RIP2 may improve outcomes in advanced breast cancer patients, in which it is overexpressed.

## Introduction

Receptor-interacting protein kinase 2 (RIP2, also known as RIPK2, RICK and CARDIAK) is a serine/threonine/tyrosine kinase with a carboxy-terminal caspase activation and recruitment domain (CARD) known for its role in inflammation and immunity [1-3]. RIP2 association with the TNF receptor (TNFR) causes direct activation of NF-kappa B and induction of apoptosis [4-6]. We had previously demonstrated an unrecognized role of RIP2 in breast cancer and as a potential chemosensitizer [7]. Here we investigate the functional significance of RIP2 expression in breast cancer.

RIP2 has been associated with activation of the NF-kappa B, c-Jun N-terminal kinase (JNK), extracellular signal-regulated kinase (ERK), and mitogen-activated protein kinase (p-38) pathways [3,6,8,9]. Involvement in metastasis has been implicated in all of these pathways. NF-kappa B has been shown to be important for promoting migration and metastasis [10] and upregulating the expression of matrix metalloproteinases, urokinase-type plasminogen activator, and cytokines in highly metastatic cancer cell lines [11-13]. In contrast, although JNK is important in inflammation, proliferation, and apoptosis, it also regulates cell migration by maintaining the labile adhesions required for rapid cell migration [14,15]. In addition, both ERK [16] and p-38 [17] have been shown to be involved in tumor cell migration.

In this study, we found that *RIP2* overexpression is most significant in triple-negative breast cancer (TNBC) and that *RIP2* expression correlates with worse progression-free survival (PFS). *RIP2* is an independent prognostic biomarker in multivariate analysis. mRNA fluorescence *in situ* hybridization (FISH) analysis of patients with locally advanced breast cancer and extensive lymph node metastases demonstrates increased *RIP2* expression compared to patients with limited lymph node metastasis. When *RIP2* expression is knocked down in MDA-MB-231, Htb126, SUM149PT, MCF7, and T47D breast cancer cells, there is significantly decreased migration as demonstrated by functional assays *in vitro*. Furthermore, *RIP2* knockdown decreases tumor cell growth during chemotherapy *in vivo* and reduces lung metastases from MDA-MB-231 xenografts. We found that *RIP2* expression regulates NF-kappa B and JNK activation in breast cancer cell lines. In addition, PP2, an RIP2 small molecule chemical inhibitor, decreases JNK activation and leads to decreased migration *in vitro*. Together, our findings demonstrate a novel role for RIP2 as a kinase involved in the migration of TBNC cells and in selected estrogen receptor (ER)-positive breast cancer cells.

## Methods

### Cells and chemicals

MDA-MB-231 and Htb126 cells were cultured in basal medium supplemented with 10% fetal calf serum and were originally purchased from American Type Culture Collection (ATCC, Manassas, VA, USA) and kindly provided by M White, Department of Cell Biology, University of Texas Southwestern Medical School, Dallas, TX. SUM149PT was purchased from Asterand (Detroit, MI, USA) and grown as per supplier’s instructions. MCF7, Htb126, and HCC1428 were purchased from ATCC and grown per supplier’s instructions. PP2 was obtained from Tocris Bioscience (Bristol, UK) and used at the indicated concentrations.

### Expression array analysis and statistics

The Cancer Genome Atlas (TCGA) breast cancer expression data and clinical annotations were downloaded from the TCGA website [18]. Five other publically available breast cancer expression datasets [19-21] were separately normalized then pooled for analysis (n = 946) as previously described [7]. Data were downloaded from GEO [22] [GSE3494 [19]; GSE6532 [20]; GSE1456 [23]; GSE7390 [24]] and from NKI [25]. These datasets were chosen for having clinical annotations that include ER status, axillary lymph node involvement, PFS and/or overall survival information. Briefly, probes were mapped using Entrez identifiers and Human Genome Organization gene symbols and then averaged for gene-level analysis. Missing values were imputed using nearest neighbor averaging. All data analysis was conducted using tools in R/Bioconductor. Datasets were merged and standardized by scaling the columns, then the rows, and finally checked using principal component analysis (PCA) as previously reported [7]. Triple-negative samples were predicted from the annotated ER-negative samples using the subtyping tool, TNBCtype [26]. Univariate and multivariate Cox regression with Pearson correlation coefficient was performed using the R survival package version 2.36-14. Wald test *P*-values are reported for univariate Cox proportional hazards model; local Wald and overall log-rank *P*-values are reported for the multivariate model using Cox proportional hazards regression model. Survival curves were evaluated by Kaplan-Meier estimators with log-rank *P*-values reported. The median expression of *RIP2* was used to split each dataset into two cohorts to examine expression patterns in samples that belong to the four clinical subtypes of breast cancer. *P*-values within boxplots were determined using the Welch two-sample *t*-test.

### Xenografts and human tissues

We injected 2,000,000 MDA-MB-231 cells into each dorsal flank subcutaneously in 6-week old NOD/SCID mice. For primary tumor growth experiments, sh-control and shRIP2 cells were injected into contralateral flanks. For lung metastasis studies, either sh-control or shRIP2 cells were injected into each mouse. Four weekly IP injections of docetaxel (5 mg/kg) were administered starting at 7 days after xenograft placement. Tumor volumes were measured weekly and were calculated as:

$$\left({\text{Width}}^{2}*\text{Length}\right){\mathrm{mm}}^{2}/2.$$

At 10 weeks after xenograft placement, primary tumors and lungs were harvested and fixed in formalin for paraffin embedding. A mid-sagittal cut was made to each lung. Two sections per lung (200 microns apart) were mounted to glass slides and stained with hematoxylin and eosin (H&E) (Thermo Scientific, Kalamazoo, MI, USA), and imaged with Zeiss Axioskop2Plus (Thornwood, NY, USA). All animal work was approved by University of Texas Southwestern (UTSW) Institutional Animal Care and Use Committee (IACUC) and performed per institutional guidelines. De-identified primary human breast tumors were obtained from the UTSW Tissue Repository where tissues were originally obtained with patient consent and used with approval from UTSW Institutional Review Board.

### Plasmids and transfections

pGIPZ RIP2 lentiviral shRNA was obtained from Open Biosystems (clone V2LHS_17021, Thermo Scientific, Inc. Pittsburgh, PA, USA). siRNAs designated as #D and #J were obtained from Dharmacon (clones D-003602-01-0005 and J-003602-09-0005, respectively from Thermo Scientific) and used with Lipofectamine RNAi Max reagent (Invitrogen, Grand Island, NY, USA) as per manufacturer’s protocol. pcDNA4 Omni-tagged RIP2 was kindly provided by D Abbott [27] (Department of Pathology, Case Western Reserve University, Cleveland, OH, USA) [28]. cDNA transfections were performed with Lipofectamine LTX reagent (Invitrogen) as per manufacturer’s protocol.

### Viral transductions and stable selections

For lentivirus production, 1 μg of pGIPZ-shRNA plasmid together with 1 μg of helper plasmids (0.4 μg pMD2G and 0.6 μg psPAX2) were transfected into 293FT cells with Effectene reagent (Qiagen, Valencia, CA, USA). Viral supernatants were collected 48 hours after transfections and cleared through a 0.45-μm filter. Cells were infected with viral supernatants containing 4 μg/mL polybrene (Sigma, St. Louis, MO) and selected with puromycin for 7 days.

### Western blot analysis and immunofluorescence

Total cell lysates were prepared by harvesting cells in Laemmli SDS reducing buffer. Protein concentrations were measured using a Pierce BCA protein assay kit (Thermo Scientific, Rockford, IL, USA), resolved on an 8% to 10% polyacrylamide gel, and transferred to a polyvinylidine fluoride membrane. The following antibodies are from Cell Signaling (Beverly, MA, USA): glyceraldehyde-3-phosphate dehydrogenase (GAPDH) (2118), p-JNK (4668), JNK (9258), p-p38 (4511), p38 (8690), p-NFkappa (3031), NFkappa (8242), p-ERK1/2 (4377), ERK 1/2(4695), p-RIP2 (4364). RICK (RIP2) antibody (sc-136059) was from Santa Cruz Biotech (Dallas, TX, USA). Focal adhesion kinase (FAK) (ab40794) and p-FAK (ab4803) antibodies were from Abcam (Cambridge, MA). Detection of peroxidase activity from HRP-conjugated antibodies was done with SuperSignal West Femto Maximum Sensitivity Substrate (Thermo Scientific, Rockford, IL, USA). Images were captured with the G:BOX F3 with GeneSys software (SynGene, Frederick, MD). Alexa Fluor 568 anti-rabbit secondary antibody (Invitrogen) was used for immunofluorescence and images were taken at 100× with Zeiss Axiovert 200M (Thornwood, NY, USA).

### Scratch/wound healing assay and Matrigel™ invasion assays

Cells were grown in normal growth media to monolayer confluence in 6-well tissue culture plates. They were then treated with mitomycin C (10 μg/mL) (Sigma, St. Louis, MO, USA) for 2 hours prior to scratching with a 1-mL pipette tip. A perpendicular pen mark on the tissue culture plate was used to mark the place for repeated imaging with an EVOS microscope (AMG/Life Technologies, Grand Prairie, TX, USA). For Matrigel™ invasion assays, cells were serum-starved for 16 hours, then 100,000 cells were seeded in the top chamber, in serum-free media, of 24-well invasion chambers, 8-μm pores (BD Biosciences, San Jose, CA, USA) with growth media in bottom chamber. Cells are allowed to migrate for 16 hours with processing procedure per manufacturer’s protocol. Hoechst 33342 (Sigma, St. Louis, MO, USA) was used to stain nuclei for imaging at 20× with the Zeiss Axiovert 200M and quantification with ImageJ (NIH, Bethesda, MD, USA).

### Fluorescence in situ hybridization

Stellaris™ mRNA FISH probes against RIP2, CAL Fluor Red 610, was obtained from Biosearch Technologies (Novato, CA, USA) and hybridization performed as per manufacturer’s protocol. Images were obtained with Deltavision (Applied Precision, Issaquah, WA, USA) and quantification was with ImageJ.

## Results

### *RIP2* overexpression correlates with triple-negative status and poor progression-free survival in breast cancer

**Figure 1:**
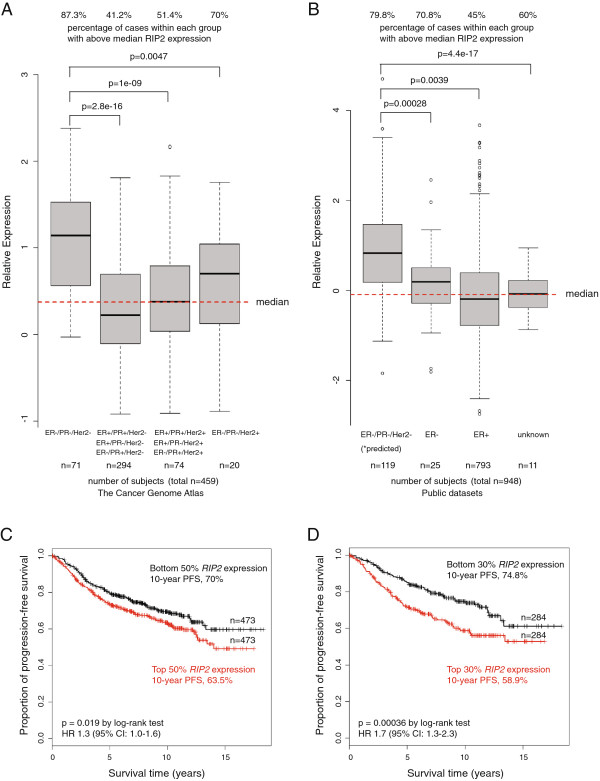
**Distribution of high- and low- *RIP2 *expressions (dichotomized at the median) of the Cancer Genome Atlas (A) and public breast cancer expression array datasets (B) (see Methods) within each designated clinical subtype of breast cancer.** Survival curves using Kaplan-Meier estimators using *RIP2* as a biomarker (dichotomized about the median in **C** and using only top and bottom 30% of all patients in **D** for progression-free survival (PFS) with log-rank *P*-values reported. Red denotes higher expression and black denotes lower expression. HR, hazard ratio.

**Figure 2:**
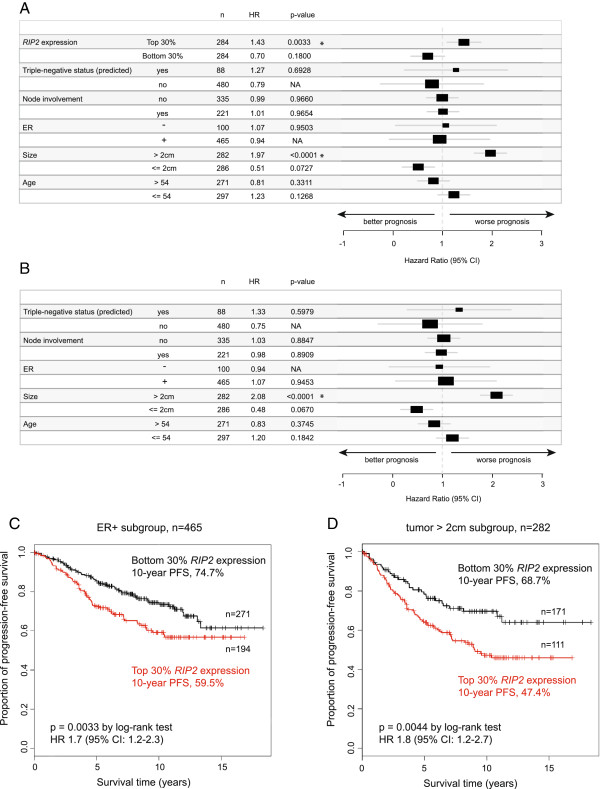
**Multivariate Cox regression analysis using various clinical features with (A) and without (B) *RIP2 *expression for progression-free survival (PFS).** *P <0.05. Kaplan-Meier estimator using top and bottom 30% *RIP2* expression to segregate ER-positive **(C)** and tumor >2 cm patient cohorts **(D)**. HR, hazard ratio; n, number of patients.

We had previously shown that KIF14, a protein-protein interactor of RIP2 [29], is significantly over-expressed in triple-negative breast cancer. We examined TCGA [30] to see if *RIP2* overexpression is also more common among triple-negative primary breast cancers. TBNCs, even though representing only 15% (71/459) of total cases, have a higher proportion of high *RIP2* expression (Figure 1A, 87.3%, left side, ER-/PR-/Her2-) relative to the other clinical subtypes (Figure 1A, middle and right side). We next tested if high-*RIP2* expression is also over-represented in other datasets. We chose five large, publicly available breast cancer expression array datasets that have adequate clinical annotations including ER status, axillary lymph node involvement, progression-free and/or overall survival data (n = 946 for PFS and n = 652 for overall survival, see Methods and as previously described [7]). As Her2 status is not annotated in these other datasets, we used the annotated ER-negative samples and obtained predicted triple-negative status from a validated public software platform TNBCtype [27]. Here, we also found that high-*RIP2* expression is over-represented in the (predicted) triple-negative subgroup compared to others (see Figure 1B). *RIP2* expression correlated to PFS but not overall survival outcomes analyzed by univariate Cox regression (*P* = 1.247e-4 and *P* = 0.281, respectively). Kaplan-Meier analysis of PFS when expression values were dichotomized at the median demonstrated the significant prognostic value of *RIP2* expression (Figure 1C, red line, high levels of *RIP2* versus black, low level of *RIP2*). The significance increased when patients in the top versus the bottom 30% of *RIP2* expression were compared (Figure 1D). As *RIP2* was highly correlated with triple-negative status, we examined this subgroup of patients (predicted from TNBCtype, n = 119; see Methods) but did not find that *RIP2* expression within the TNBC cohort correlated with PFS when expression values were dichotomized at the median (*P* = 0.35, hazard ratio (HR) 0.8, 95% CI 0.4, 1.3) or when only the top versus bottom 30% of *RIP2* expression were used (*P* = 0.68, HR 1.3, 95% CI 0.4, 4.1). On the other hand, multivariate analysis using Cox proportional hazards regression analysis using *RIP2* expression and clinical features (triple-negative status, axillary lymph node involvement, ER status, size, and age) indicated that *RIP2* is an independent prognostic biomarker (*P* = 0.0033, HR 1.43, 95% CI 1.10, 1.77) in addition to tumor size greater than 2cm (*P* <0.0001, HR 1.97, 95% CI 1.65, 2.29) (multivariate analysis with and without *RIP2* expression in Figure 2A and B, respectively). Other clinical factors such as axillary lymph node involvement, ER status, and age were not independent prognostic factors for PFS on multivariate analysis although triple-negative status and younger age (younger than median age) trended towards worse survival (Figure 2A and B). To further validate *RIP2* expression as an independent prognostic biomarker, we evaluated the prognostic value of *RIP2* expression for only ER-positive patients (n = 465). High and low *RIP2* expression (top versus bottom 30% of the overall median expression value of the combined dataset) segregated patients into a high- and low-risk group, respectively (Figure 2C). There was no direct correlation between *RIP2* expression and tumor size (*r* = 0.173, Pearson’s correlation, data not shown). However, within the larger tumor cohort (size >2 cm), higher *RIP2* expression is also prognostic of worse PFS (Figure 2D).

### *RIP2* expression affects migration and invasion

**Figure 3:**
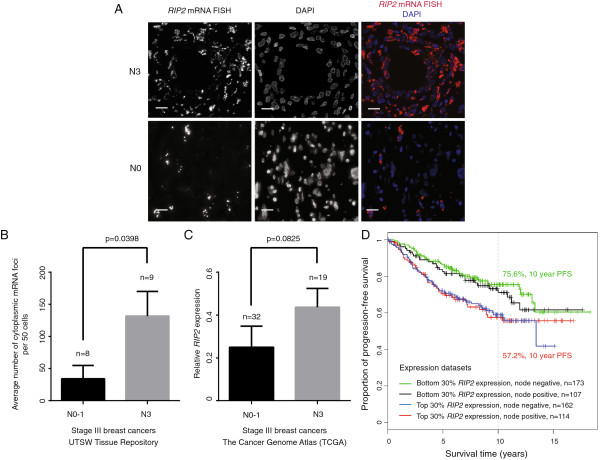
***RIP2 *mRNA expression in primary tumors suggests correlation with extensive axillary lymph node invasion in stage III breast cancer. (A)***RIP2* mRNA fluorescence *in situ* hybridization (FISH) in primary breast tissue of a representative patient with N3 (top panels) and N0 (bottom panels) breast cancer. Scale bar = 20 μM. **(B)** Average number of cytoplasmic mRNA foci per 50 cells was used to compare the group of stage-III patients from the University of Texas Southwestern Medical Center (UTSW) Tissue Repository with extensive (N3) versus limited (N0, N1) lymph node involvement. Average of three imaging fields per case with error bars representing standard error of the mean (SEM). **(C)** Relative *RIP2* expression of stage-III patients from The Cancer Genome Atlas (TCGA) with N3 versus N0/N1 disease. Statistics per two-sided Student *t*-test for **B**, **C**. **(D)** Kaplan-Meier estimator to evaluate *RIP2* expression and lymph node status as prognostic indicator in the combined public expression array datasets. PFS, progression-free survival.

As tumor metastasis is the main cause of morbidity and mortality, we investigated the effect of *RIP2* expression on cell migration. Metastasis of breast cancer primarily occurs through the regional lymphatic system with the extent of axillary lymph node involvement as a key prognostic factor for the disease. We obtained 17 cases of stage-III breast cancers with either little or extensive axillary lymph node involvement, from the University of Texas Southwestern (UTSW) Tissue Repository. Specifically, we evaluated *RIP2* expression by mRNA FISH in stage-III breast cancer cases with either fewer than four axillary lymph nodes (N0, N1) or with significant axillary lymph node involvement (N3 or tumor metastasis in more than 10 axillary lymph nodes, infraclavicular lymph nodes, supraclavicular lymph nodes, or clinically detected ipsilateral internal mammary lymph nodes along with other axillary lymph nodes). We found that *RIP2* expression appears to be more prominent in breast cancers that have significant axillary lymph node spread (Figure 3A,B). The differential *RIP2* expression is not due to triple-negative status alone (percentage of triple-negative patients in N0/N1 groups was 33% and in N3 group 25%) and suggests a specific role for *RIP2* in migration.

We interrogated TCGA for stage-III patients with N0 to N1 versus N3 disease and found a similar trend of higher expression in N3 disease, although the *P*-value for the two-sided *t*-test was not significant (*P* = 0.825, see Figure 3C). This needs to be examined further, especially in a larger dataset of stage-III patients.

Although the public datasets we examined lack adequate annotation for stage determination (only binary information given for lymph node status), we evaluated how lymph node status may affect *RIP2* expression as a prognostic indicator (evaluating patients in the top versus bottom 30% of *RIP2* expression) and found that high *RIP2* expression with node positivity correlated with significantly worse PFS compared to low *RIP2* expression with node negativity (log-rank *P* = 0.0042, HR 1.9, 95% CI 1.2, 2.8; Figure 3D).

**Figure 4:**
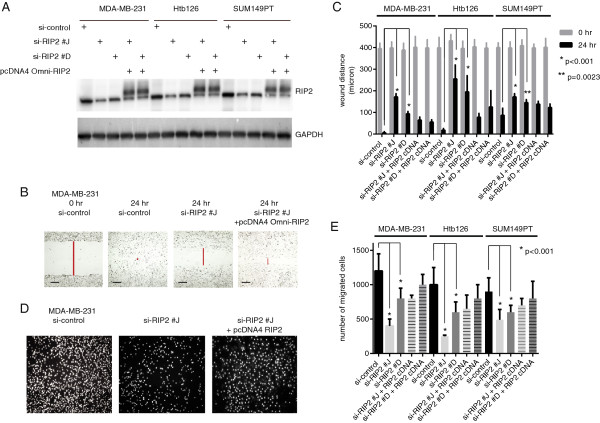
***RIP2 *knockdown decreases migration and invasion in triple-negative breast cancer *(*TNBC) cell lines. (A)** Western blot showing relative protein expressions of *RIP2* knockdown (si-RIP2 #J and si-RIP2 #D) and pcDNA4 Omni-tagged RIP2 in three TBNC cell lines. **(B)** Representative scratch/wound healing assay images for MDA-MB-231 cells. **(C)** Quantification of scratch/wound healing assay results for three triple-negative cell lines and error bars represent standard error of the mean (SEM). Data represent results from three separate experiments. Statistics per Student *t*-test as indicated. Scale bar = 100 μM. **(D)** Representative Matrigel™ invasion assays from MDA-MB-231 cells at 16 hours. **(E)** Quantification of Matrigel™ invasion assay results for three triple-negative cell lines with error bars representing SEM. Columns represent results from triplicates repeated in three separate experiments. Statistics per Student *t*-test.

To further evaluate the correlation of *RIP2* expression with migration or invasion *in vitro*, we generated *RIP2* knockdown using two short-interfering RNAs (siRNAs), designated si-RIP2 #J and si-RIP2 #D, in the TBNC cell lines MDA-MB-231, Htb126, and SUM149PT (Figure 4A, see Methods). Among available TBNC cell lines, we chose these triple-negative cell lines that have basal or mesenchymal/stem-like characteristics, as they represent subtypes of breast cancer with poor prognosis and no available targeted therapies [31]. Both the scratch/wound healing and Matrigel™ transwell invasion assays showed that decreased *RIP2* expression led to decreased migration. Importantly, reconstituted *RIP2* expression (pcDNA4 Omni-RIP2) largely restored migratory ability in all three triple-negative breast cancer cell lines (Figure 4B-E), indicating that the migration deficiency is unlikely to be an off-target effect of our siRNAs and that RIP2 has a clear role in migration.

**Figure 5:**
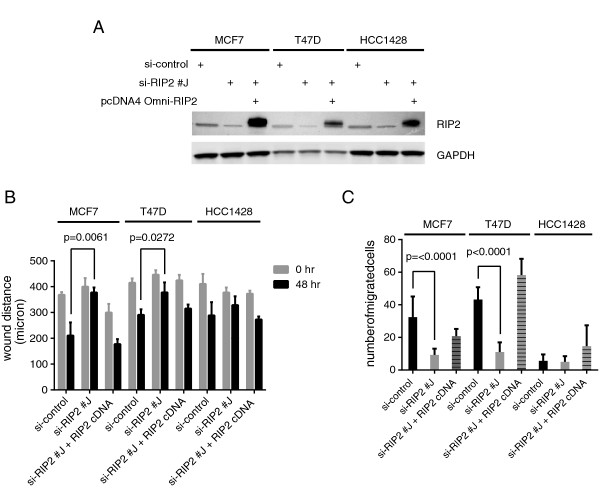
***RIP2 *knockdown decreases migration and invasion in certain estrogen receptor (ER)-positive cell lines. (A)** Western blot showing relative protein expressions of RIP2 knockdown (siRIP2 #J) and pcDNA4 Omni-tagged RIP2 in 3 ER-positive breast cancer cell lines. **(B)** Quantification of scratch/wound healing assay results for three triple negative cell lines and error bars represent standard error of the mean (SEM). Data represent results from two separate experiments. Statistics per Student *t*-test as indicated. **(C)** Quantification of Matrigel™ invasion assay results for three ER-positive cell lines with error bars representing SEM. Columns represent results from triplicates repeated in two separate experiments. Statistics per Student *t*-test.

As our *in silico* survival studies indicated a potential role for *RIP2* in ER-positive breast cancer, we then tested if *RIP2* expression affects migratory ability of ER-positive breast cancer cell lines MCF7, T47D, and HCC1428 (Figure 5A,B,C). ER-positive cell lines migrate less and slower in both the scratch/wound healing (Figure 5B) and the Matrigel™ invasion (Figure 5C) assays compared to the TNBC cell lines (Figure 4C,E). Knockdown of *RIP2* decreases the migratory ability of MCF7 and T47D cells but not HCC1428 cells (Figure 5B,C).

### *RIP2* expression affects chemosensitivity *in vivo* and tumor cell migration

**Figure 6:**
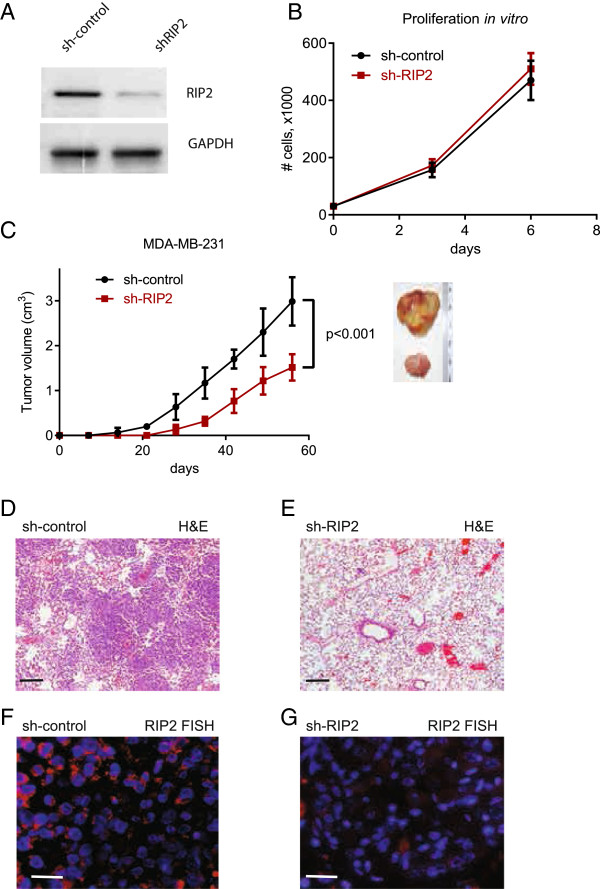
***RIP2 *knockdown does not cause decrease in proliferation but does cause decreased tumor growth and lung metastasis *in vivo *with docetaxel treatments. (A)** Western blot showing relative RIP2 expression in sh-control and sh-RIP2 MDA-MB-231 cells. **(B)** Relative proliferation rate of sh-control and sh-RIP2 MDA-MB-231 cells *in vitro*. **(C)** Tumor volume at various time points after xenograft placements. Images are representative of final tumors at 10 weeks for sh-control (top) and sh-RIP2 (bottom). Statistics per Student *t*-test at final time point recorded. Representative H&E stains of lung sections at 10 weeks are shown **(D** and **E)**. Scale bar represents 100 μm. Representative RIP2 fluorescence *in situ* hybridization (FISH) images of lung sections are shown in **(F)** and **(G)**. Red, RIP2 mRNA signal. Blue, 4',6-diamidino-2-phenylindole (DAPI) nuclear stain. Scale bar represents 25 μm.

We next investigated the *in vivo* effect of RIP2 knockdown using mouse xenograft studies. We injected equal numbers of short-hairpin (sh) RNA, sh-control and sh-RIP2 stably expressed in MDA-MB-231 cells (Figure 6A) into contralateral dorsal flanks of immunodeficient NOD-SCID mice. As RIP2 knockdown by itself has little effect on cell growth *in vitro* (Figure 6B), we used docetaxel (one of the most common chemotherapies given for breast cancer) to determine if *RIP2* knockdown would chemosensitize tumor cells to docetaxel. *RIP2* knockdown cells show a significant decrease in tumor mass after docetaxel treatments compared to non-silencing sh-control MDA-MB-231 cells injected into contralateral dorsal flanks (Figure 6C).

We also examined the metastatic potential of sh-control and shRIP2 MDA-MB-231 cells (these were originally derived from a malignant pleural effusion) to the lungs. Strikingly, after four weekly doses of docetaxel, lung metastases were noticeably present in all animals that had only sh-control xenografts (n = 3/3 animals) (Figure 6D) with large areas where the tumor cells replaced the lung parenchyma at the time of sacrifice (2.5 months after xenograft placement). In contrast, no obvious lung metastases were present in the animals with sh-RIP2 only xenografts (n = 6/6 animals) (Figure 6E). *RIP2* FISH of the lungs showed that sh-control cells to the lung retain high *RIP2* expression whereas the small amount of sh-RIP2 cells that metastasized to the lungs continued to have low *RIP2* expression (Figure 6F and G, respectively). The significant difference in the presence of lung metastasis may in part be related to the differences seen in the primary tumor size in response to chemotherapy. Additional studies *in vivo* will be needed to more fully ascertain the role of RIP2 in metastasis.

### RIP2 is an activator of the NF-kappa B and JNK pathways

**Figure 7:**
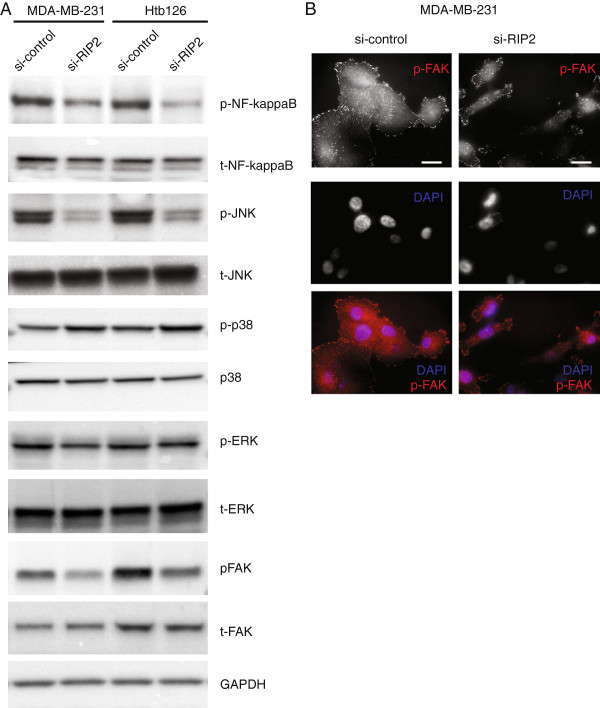
**RIP2 affects activation of NF-kappaB and JNK pathways. (A)** Western blots showing relative expressions of various proteins in si-control and si-RIP2 cells in 2 triple negative breast cancer cell lines. **(B)** pFAK immunofluorescence of MDA-MB-231 cells show less abundant pFAK staining around si-RIP2 cells relative to si-control cells. Scale bar represents 10 μm.

To understand specific mechanisms underlying the migration defect seen with RIP2 knockdown in TBNC cells, we evaluated known pathways downstream of RIP2, including the NF-kappa B, ERK, JNK, and p-38 pathways [32]. In MDA-MB-231 and Htb126 cells, two of the cell lines with the greatest migration defects in our *in vitro* assays, *RIP2* knockdown is associated with decreased phosphorylation of NF-kappa B and JNK but not p38 or ERK (Figure 7A). A slight increase in the phosphorylation of p38 can be observed (Figure 7A). Both JNK and NF-kappa B are associated with defects in adhesion [14,15,33]. *Focal adhesion kinase* (*FAK*) has been well-established as a gene significant for cancer cell migration and metastasis (reviewed in [34]) and is currently being used either as a drug target or as a biomarker in various solid tumors (see *Clinicaltrials.gov*). We therefore evaluated the expression of p-FAK to see if RIP2 expression correlates with this well-known biomarker for migration and metastasis. We found that although sh-control MDA-MB-231 cells have ample focal adhesions around the cells, RIP2 knockdown cells have fewer focal adhesions as reflected by decreased p-FAK protein (Figure 7A) and less continuous p-FAK staining around the cells (Figure 7B).

### RIP2 kinase activity is necessary for JNK activation but not NF-kappa B

**Figure 8:**
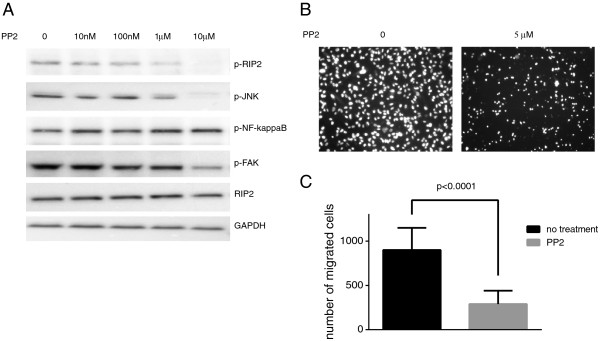
**Chemical inhibition of RIP2 leads to decreased JNK and FAK activation. (A)** Western blots showing relative expressions of various proteins in MDA-MB-231 cells treated with indicated doses of PP2. **(B)** Representative Matrigel™ invasion assays from MDA-MB-231 cells at 16 hours when treated with indicated doses of PP2. **(C)** Quantification of Matrigel™ invasion assay results for MDA-MB-231 cells treated with PP2 with error bars representing standard error of the mean (SEM). Columns represent results from two separate experiments done in triplicates. Statistics per Student *t*-test.

PP2 is a known small-molecule chemical inhibitor of RIP2 [35] and the Src family of tyrosine kinases [36]. We found that at 1 μM PP2 has a noticeable effect on the amount of RIP2 phosphorylation in MDA-MB-231 cells. At the same concentration of PP2, there was a correlated decrease in the amount of phosphorylation of JNK and FAK but not NF-kappa B (Figure 8A), implicating JNK activation as a key downstream effector for RIP2-mediated migration in MDA-MB-231 cells. As expected, there was a significant decrease in the number of cells that migrated in the Matrigel™ transwell invasion assay (Figure 8B,C) when cells were treated with PP2.

## Discussion

To determine immediate actionable targets from our previous chemosensitivity screen, we have identified *RIP2* as a gene important for breast cancer cell metastasis. RIP2 is a kinase with known central roles in inflammation and immunity. We have demonstrated a novel role for RIP2 in the metastatic potential of multiple breast cancer cells with high *RIP2* expression both *in vitro* and *in vivo* and shown that it is a target for drug treatment in cancer therapy.

Although *RIP2* is over-expressed more often in TNBC than other clinical subtypes, we demonstrated in the present study that *RIP2* expression is an independent prognostic biomarker. Although high *RIP2* expression correlated with triple-negative status, it was not a mere surrogate of triple-negative status, as high *RIP2* expression also correlated with poor survival in ER-positive breast cancers and those with tumor size >2 cm. However, although RIP2 may also be a target for other types of breast cancer (that is, ER-positive), its high expression within the triple-negative subset identifies this cohort as the most likely to show immediate benefit from targeted treatment against RIP2.

Axillary lymph node metastasis in primary breast cancers has long been used clinically as a poor prognostic indicator [37] but there remains controversy about whether axillary lymph node metastasis reflects lead-time bias or true tumor biology (that is, metastatic potential or aggressiveness) [37-39]. Multivariate analysis of our combined expression array datasets did not reveal lymph node status to be prognostic for PFS although we found that *RIP2* mRNA expression correlated with extensive axillary lymph node invasion in stage-III breast cancer. This suggests that a combination of lymph node status and *RIP2* expression may prove to be of prognostic utility in the clinical setting. However, studies involving larger samples are needed to elucidate the role of *RIP2* in axillary lymph node spread.

Here we provided *in vitro* evidence for the role of *RIP2* in migration and invasion in TNBC and selected ER-positive breast cancers. The effect of *RIP2* knockdown in ER-positive cell lines, although significant for MCF7 and T47D, was modest compared to the effect of *RIP2* knockdown in the triple-negative cell lines. TNBC and ER-positive breast cancers have significantly different clinical courses, with TNBC generally being more likely to relapse, metastasize, and be resistant to treatment [40]. Expression array analysis of TBNC has revealed multiple subtypes, each with various potential targets [31]. Recent large clinical trials have failed to validate the clinical efficacy of various targets deemed to be important for subsets of TNBC (examples include iniparib against poly-ADP ribose polymerase [41] and bevacizumab against vascular endothelial growth factor receptor [42]). There is clearly an unmet need for additional therapeutic targets. As high expression of *RIP2* is found in approximately 80 to 90% of TNBCs and approximately 50% of ER-positive breast cancers, RIP2 kinase is an attractive novel therapeutic target for the majority of TNBC and selected ER-positive breast cancers that have upregulated RIP2.

We have found that *RIP2* knockdown correlates with decreased phosphorylation of JNK and NF-kappa B in TNBC. Both of these pathways have well-known roles in cancer progression. In our current studies, we observed a slight increase in the phosphorylation of p38, suggesting a potential compensatory effect. JNK and p38 integrate signals involved in proliferation, differentiation, survival and migration (reviewed in [43]). Conversely, NF-kappa B activation by RIP2 has been shown to be secondary to interactions from a multimeric complex of proteins and not directly from RIP2 kinase activity [44]. Our results suggest that although RIP2 may be a crucial member of the complex that controls NF-kappaB activation, its kinase activity is not necessary for NF-kappa B activation. However, the kinase activity of RIP2 appears to be important for the activation of JNK. Further experiments on the mechanism of RIP2 activation of JNK and NF-kappa B, along with potential compensatory alterations in other pathways are necessary.

## Conclusions

We previously found that *RIP2* knockdown increases chemosensitivity [7]. Here, we demonstrated that RIP2 has a role in facilitating metastasis and is an independent prognostic marker for breast cancer. Its association with human breast tumors and patient outcome warrants further investigation. We have identified *RIP2* as a gene that is upregulated in the majority of TBNCs and likely contributes to the metastatic process.

## Abbreviations

ATCC: American Type Culture Collection; ER: estrogen receptor; ERK: extracellular signal-regulated kinase; FAK: Focal adhesion kinase; FISH: fluorescence *in situ* hybridization; GAPDH: glyceraldehyde-3-phosphate dehydrogenase; GEO: Gene Expression Omnibus; H&E: Hematoxylin and eosin; IP: intraperitoneal; JNK: c-Jun N-terminal kinase; p-38: mitogen-activated protein kinase; PFS: progression-free survival (the time elapsed between treatment initiation and tumor progression or death from any cause with censoring of patients who are lost to follow-up); PP2: 3-(4-chlorophenyl) 1-(1,1-dimethylethyl)-1*H*-pyrazolo(3,4-*d*)pyrimidin-4-amine; RIP: Receptor-interacting protein kinase 2; SEM: standard error of the mean; shRNA: short-hairpin RNA; siRNA: small-interfering RNA; TCGA: The Cancer Genome Atlas; TNBC: triple-negative breast cancer; TNF: tumor necrosis factor; UTSW: The University of Texas Southwestern Medical Center in Dallas.

## Competing interests

The authors declare that they have no competing interests.

## Authors’ contributions

SMS: conception and design, development of methodology, acquisition of data, analysis and interpretation of data, writing of the manuscript. CC: development of methodology, acquisition of data, manuscript revision. KB: development of methodology, acquisition of data, manuscript revision. GJ: acquisition of data, manuscript revision. SLB: acquisition of data, manuscript revision. GFL: development of methodology, acquisition of data, manuscript revision. WEW: analysis and interpretation of data, manuscript revision. JWS: analysis and interpretation of data, manuscript revision. All authors read and approved the final manuscript.
